# Genome-Wide Identification and Analysis of the Ascorbate Peroxidase (APX) Gene Family of Winter Rapeseed (*Brassica rapa* L.) Under Abiotic Stress

**DOI:** 10.3389/fgene.2021.753624

**Published:** 2022-01-21

**Authors:** Li Ma, Weiliang Qi, Jing Bai, Haiyun Li, Yan Fang, Jia Xu, Yaozhao Xu, Xiucun Zeng, Yuanyuan Pu, Wangtian Wang, Lijun Liu, Xuecai Li, Wancang Sun, Junyan Wu

**Affiliations:** ^1^ State Key Laboratory of Aridland Crop Science/College of Agronomy, Gansu Agricultural University, Lanzhou, China; ^2^ College of Agriculture and Forestry, Longdong University, Qingyang, China; ^3^ Zhangye Academy of Agricultural Sciences, Zhangye, China; ^4^ Collaborative Innovation Center for Western Ecological Safety, Lanzhou University, Lanzhou, China; ^5^ College of Agronomy and Biotechnology, Hexi University, Zhangye, China

**Keywords:** APX gene family, cold resistance, *Brassica rapa*, *Brassica napus*, expression analysis

## Abstract

Winter *Brassica rapa* (*B. rapa*) is an important oilseed crop in northern China, but the mechanism of its cold resistance remains unclear. Ascorbate peroxidase (APX) plays important roles in the response of this plant to abiotic stress and in scavenging free radicals. In this study, the roles of APX proteins in the cold response and superoxide metabolism pathways in rapeseed species were investigated, and a comprehensive analysis of phylogeny, chromosome distribution, motif identification, sequence structure, gene duplication, and RNA-seq expression profiles in the *APX* gene family was conducted. Most *BrAPX* genes were specifically expressed under cold stress and behaved significantly differently in cold-tolerant and weakly cold-resistant varieties. Quantitative real-time-PCR (qRT-PCR) was also used to verify the differences in expression between these two varieties under cold, freezing, drought and heat stress. The expression of five *BrAPX* genes was significantly upregulated in growth cones at 3 h of cold stress, while their expression was significantly lower at 24 h than at 3 h. The expression of Bra015403 and Bra003918 was significantly higher in “Longyou-7” growth cones than in other treatments. Five *BrAPXs* (Bra035235, Bra003918, Bra033040, Bra017120, and Bra031934) were closely associated with abiotic stress responses in *B. rapa*. These candidate genes may play important roles in the response of *B. rapa* to low temperature stress and provide new information for the elucidation of the cold resistance mechanism in *B. rapa*.

## Introduction

*Brassica rapa* (*B. rapa*) (2n = 20, AA) comprises several subspecies that provide human nutrition in the form of leafy, root and stem vegetables and edible oils. It also represents the origin of the genome of *Brassica* “A” genome and contributes to the *Brassica* allopolyploid oil crops *Brassica napus* (*B. napus*) (AACC) and *Brassica juncea* (*B. juncea*) (AABB) (Cheng et al., 2014). Thus, *B. rapa* has great potential as a model for genomic and evolutionary studies of species in the genus *Brassica*. In the last decade, an increasing number of genomic resources for *B. rapa* have become available (Tong et al., 2013; Zhang et al., 2015). *B. rapa* is one of the major oil crops in China, and the yield and oil content of *B. rapa* from northern China are higher than those of spring rapeseed and other oil crops (Sun et al., 2007). Overwintering has been the main factor limiting the development of *B. rapa* production. In most regions in China north of 35° north latitude, extreme winter temperatures are below −20°C, cumulative negative air temperature is less than −500°C‧a, and winter rainfall is below 30 mm (Sun et al., 2016; Ma et al., 2019b). Unfavourable factors such as severe cold and dry winters in northern China, which make it difficult for crops other than winter wheat to overwinter (Cai and Jiang, 2011). According to the overwintering rate and cold resistance performance, the cold resistance of *B. rapa* can be divided into four types: ultra-cold resistance, strong cold resistance, cold resistance and weak cold resistance varieties. In the areas east of the Wushaoling Mountain, most cold-resistant varieties can be overwintered, but in the Hexi Corridor, only ultra-cold-resistant and strong cold-resistant varieties can be overwintered (Sun et al., 2007). The breeding and production of *B. rapa* varieties have solved the problem of overwintering for oilseed crops and made northern cold and dry regions an important winter rapeseed production region in China, thereby increasing cropping intensity and economic return.

Reactive oxygen species (ROS) play an important role as a regulator of cellular response to environmental factors in plants, including superoxide anions (O^2−^), hydrogen peroxide (H_2_O_2_) and hydroxyl radicals (OH^−^), which destroy DNA and cellular components via lipid peroxidation and protein oxidation. Most cold-tolerant plants have evolved antioxidant defense mechanisms to defend against excessive accumulation of these ROS substances, including protective substances such as ascorbic acid, superoxide dismutase (SOD), catalase (CAT), and ascorbate peroxidase (APX) (Hernández et al., 2001; Gilroy et al., 2016). APX belongs to the peroxidase superfamily in plants and plays an important role in ROS scavenging pathways (Hodges et al., 1999; Apel and Hirt, 2004). APX is a key enzyme in the ascorbate-glutathione cycle that has evolved in plants to scavenge H_2_O_2_ from plant chloroplasts and the cytoplasm. It uses ascorbic acid as an electron donor to scavenge H_2_O_2_ produced in plants and thereby improves resistance to oxidative stress and enhances stress resistance (Winfield et al., 2010; Miura and Furumoto, 2013; Ding et al., 2019). The important role of the APX gene family in antioxidant stress has now been demonstrated in a variety of plants. Nine APX and seven GPX members have been identified in *sorghum*, and RNA-seq and quantitative real-time-PCR (qRT-PCR) analysis showed that *APX*/*GPX* genes were significantly regulated under drought stress (Akbudak et al., 2018). Antioxidant and cold resistance studies on transgenic cassava co-expressing cytoplasmic *MeCu*/*ZnSOD* and *MeAPX2* showed that *SOD* and *APX* expressed at high levels in transgenic plants scavenged ROS and activated antioxidant defense mechanisms, thus improving tolerance to cold stress (Xu et al., 2014). A study of Arabidopsis revealed that the regulation of plastid *APX* could transmit information on previous cold stress over time without the establishment of cold adaptation (Buer et al., 2016). Sato et al. (2001) cloned the promoter of the *APXa* gene from rice, found that it featured a minimum heat shock factor binding motif 5′-nGAAnnTTCn-3′, located 81 bp upstream of the TATA box, and confirmed heat shock-mediated *APX* gene expression and protection against chilling in rice seedlings. However, there are few studies on the APX regulation of cold acclimation in *B. rapa*.

In this study, we conducted a genome-wide identification of 118 family members of *APX* genes in *B. rapa* and performed a comprehensive analysis of their phylogeny, chromosome distribution, motif identification, sequence structure and gene duplication. We also used RNA-seq data to identify *APX* genes in *B. rapa* and *B. napus* expressed in response to cold stress and used qRT-PCR data to validate the specific expression of these genes under cold stress. This study provides information on the involvement of *APX* genes in the cold stress response in *B. rapa*, which will help to further elucidate the mechanism underlying strong cold resistance in *B. rapa*.

## Materials and Methods

### Plant Growth and Cold Treatments

Two *B. rapa* varieties, “Longyou-7” (Ultra-cold-resistant, AA, 2n = 20) and “Lenox” (weakly cold-resistant, AA, 2n = 20), were selected for their wide variation in cold resistance. The overwintering rates of “Longyou-7” and “Lenox” for many years have been approximately 90 and 10%, respectively, in Shangchuan Town, Yongdeng County, Lanzhou City, Gansu Province, China (103°40′ E, 36°03′ N), where the altitude is 2,150 m, the average annual temperature is 6.5°C, the average minimum temperature in the coldest month is approximately −14.6°C, the extreme low temperature is −26.5°C and the average annual precipitation is 175 mm (Sun et al., 2007; Pu Y. Y. et al., 2019). The plants were grown in a greenhouse at Gansu Agricultural University in Gansu Province, China. The seeds were obtained from the rapeseed breeding group of Gansu Agricultural University, and were selected by our team. This study complies with relevant institutional, national, and international guidelines and legislation. A total of 120 seeds were surface-sterilized in 10% H_2_O_2_ for 30 min, soaked in distilled water for 10 min, and washed three times to remove H_2_O_2_. Seeds were germinated on two layers of wet filter paper in a glass petri dish and placed in a plant incubator (22/18°C with 16 h light/8 h dark cycle, light intensity 2600 lux) for 2 days. Three uniform seedlings of each cultivar were selected, with good growth condition and consistent growth stages, and transplanted into 15-cm diameter seedling pots filled with matrix and vermiculite (volume: volume, 3:1). At the six-leaf stage, cold, freezing and heat stress treatments were carried out in incubators at 4°C, −4°C and 40°C, respectively. Drought stress treatment was applied by adding 200 ml of a nutrient solution containing 18% PEG6000 to the medium of each pot. Under a variety of abiotic stresses, the leaves of these two varieties with different cold resistance showed markedly different phenotypic characteristics as the treatment time progressed. Growth cones are considered to be the most direct site for sensing temperature changes and stresses, while roots (root length and root diameter) can show great variation in pots. Studies have shown that cold stress causes significant changes in phenotype and gene expression in rapeseed leaves and growth cones (Ma et al., 2019a; Qi et al., 2020). Therefore, leaf and growth cone sites were used for this project study. First, samples were placed in separate incubators for each treatment (22/18°C, 16 h/8 h light, 2600 lux light intensity) and left to stand for 24 h. The drought stress treatments were imposed by adding 200 ml of a nutrient solution containing 18% PEG 6000 to pots, the salt stress treatments were imposed by adding 200 ml of a nutrient solution containing 180 mM NaCl to pots. The cold and heat stress treatments were applied in an incubator set at 4 and 40°C, respectively. Leaves and growth cones were sampled at 0 h (CK), 3 h, and 24 h (same photoperiod and light intensity as above) after different abiotic stress treatments, and immediately frozen in liquid nitrogen and stored at −80°C for further analysis. For each type and duration of stress treatment, three separate pots were used as replicates (Ma et al., 2020).

### Identification and Analysis of Peroxidase Genes in *B. rapa* and *B. napus*

The HMM (hidden Markov model) file of the peroxidase domain PF00141 was downloaded from the Pfam database^1^ (Finn et al., 2016), and the genome and protein sequences of *B. rapa* and *B. napus* were downloaded from the *Brassica* database (BRAD^2^) (Cheng et al., 2011). First, the HMM profiles corresponding to the peroxidase domain sequences of *B. rapa* and *B. napus* were constructed by HMMER 3.1 software^3^ with an *E*-value ≤1^e−10^ (Mistry et al., 2013), and then SMART software^4^ (Letunic et al., 2015), NCBI-CDD^5^ and the Pfam database were used to remove redundant sequences and confirm the obtained peroxidase proteins (Lu et al., 2016; Saha et al., 2016; Marchler-Bauer et al., 2017; Akbudak et al., 2018). Finally, the final sequence file was manually selected and confirmed for follow-up experiments. The basic physicochemical properties of protein sequences were analyzed using the ExPasy site^6^ (Gasteiger et al., 2003).

### Sequence Analysis, Structural Identification and Phylogenetic Classification of *BrAPX* Genes

The conserved motifs in the *BrAPX* sequence were identified using the MEME tool (version 5.0.4^7^) with the following parameters: the maximum number of motifs was set to 10, and the optimal motif width was six to 50 amino acid residues (Bailey et al., 2009). Exon-intron structural information for the *BrAPX* genes was mapped using the Gene Structure Display Server (GSDS2.0^8^) (Hu et al., 2015). Homologous sequence alignment of APX amino acid sequences identified in *B. rapa* and *B. napus* was performed using the ClustalW program (Sievers et al., 2011), and an unrooted phylogenetic tree was generated using MEGA (version 7.0) by the maximum likelihood (ML) method with 1,000 bootstrap samples (Saitou and Nei, 1987; Kumar et al., 2016).

### Analysis of Chromosomal Distribution and Gene Duplication in *B. rapa*

Chromosomal position mapping of *BrAPX* genes was performed using MapChart software (Voorrips, 2002). Analysis and visualization of tandem duplication and segmental duplication between *B. rapa* and itself and *B. rapa* and *B. napus* was accomplished using the Multiple Collinearity Scan toolkit (MCscanX^9^) and Circos (version 0.69) (Krzywinski et al., 2009; Wang et al., 2012). KaKs Calculator (version 2.0^10^) was used to calculate synonymous (Ks) and nonsynonymous (Ka) substitutions to further characterize the variation in the *BrAPX* genes (Wang et al., 2010).

### RNA Isolation, Quantitative Real-Time PCR and RNA-Seq Analysis

Total RNA was isolated using a *SteadyPure* Plant RNA Extraction Kit (Accurate Biotechnology, AG21019, Hunan, China) following the manufacturer’s instructions and removing genomic DNA contamination. A spectrophotometer (NanoVueTM Plus, Wilmington, DE, United States of America) was used to evaluate RNA concentration and mass by determining the A_260_/A_280_ and A_260_/A_230_ ratios, respectively. First-strand cDNA was synthesized with *Evo M-MLV* RT Premix (Accurate Biotechnology, AG11706, Hunan, China) according to the instructions. qRT-PCR was performed on an ABI QuantStudio 5 (Thermo Fisher Scientific, Shanghai, China) using the SYBR® Green Premix *Pro Taq* HS qRT-PCR Kit (Accurate Biotechnology, AG11718, Hunan, China) with primers provided in Supplementary Table S1. The qRT-PCR reaction conditions were as follows: 30 s at 95°C, followed by 40 cycles of 5 s at 95°C and 30 s at 60°C, followed by 65–95°C melting curve detection. The qRT-PCR efficiency of the genes was obtained by analyzing the standard curve of cDNA gradient dilution, and the gene fragment encoding *B. rapa β-actin* RNA was used as the internal control to normalize the amount of template cDNA. Relative expression values for each gene were computed using the comparative 2^-ΔΔCT^ method with normalization to the internal control gene (Giulietti et al., 2001; Livak and Schmittgen, 2001).

Four RNA-seq libraries were used for expression analysis in the following steps 1. RNA-seq library (SRP179662) was derived from growth cones of *B. rapa* cultivars “Longyou-7” (ultra-cold resistance) and “Lenox” (weakly cold-resistant) under cold stress (22°C as a control, and 4°C for 3 and 24 h) (Ma et al., 2019a). 2. RNA-seq library (SRP211768) was derived from roots of *B. rapa* cultivars “Longyou-7” (ultra-cold resistance) and “Tianyou-2” (weakly cold-resistance) under freezing stress (22°C as a control, and −4°C for 6 h)3. RNA-seq library was derived from leaves of *B. napus* cultivars “NTS-309” (strong cold resistance) and “Tianyou-2238” (weakly cold-resistant) under cold stress (25°C as a control and 4°C for 48 h) (Qi et al., 2020). 4. RNA-seq library (SRP540905) was derived from leaves of *B. napus* cultivar “2016TS(G)10” (strong cold-resistance) under freezing stress (22°C as a control and −2°C for 1, 3, and 24 h) (Pu Y. et al., 2019). RNA-seq means from the same gene in two samples were considered statistically significant and a heat map was drawn with TBtools software^11^ when there was a fold change greater than 2 and when the adjusted *p*-value was less than 0.05 (Chen et al., 2020; Liu et al., 2020). Interacting genes/proteins were retrieved by STRING (V11.5) software^12^ to construct protein-specific interaction networks with a combined score ≥400 (medium confidence).

### Statistical Analysis

One-way analysis of variance and Duncan’s multiple range test were used to detect significant differences among the means of the plant treatment groups using SPSS 19.0 statistical software (SPSS Inc., Chicago, IL, United States of America). A *p*-value ≤ 0.05 was considered statistically significant. All results are represented as the mean ± standard error of the mean of at least three replications.

## Results

### Identification, Chromosomal Distribution, and Classification of BrAPX Genes

**FIGURE 1:**
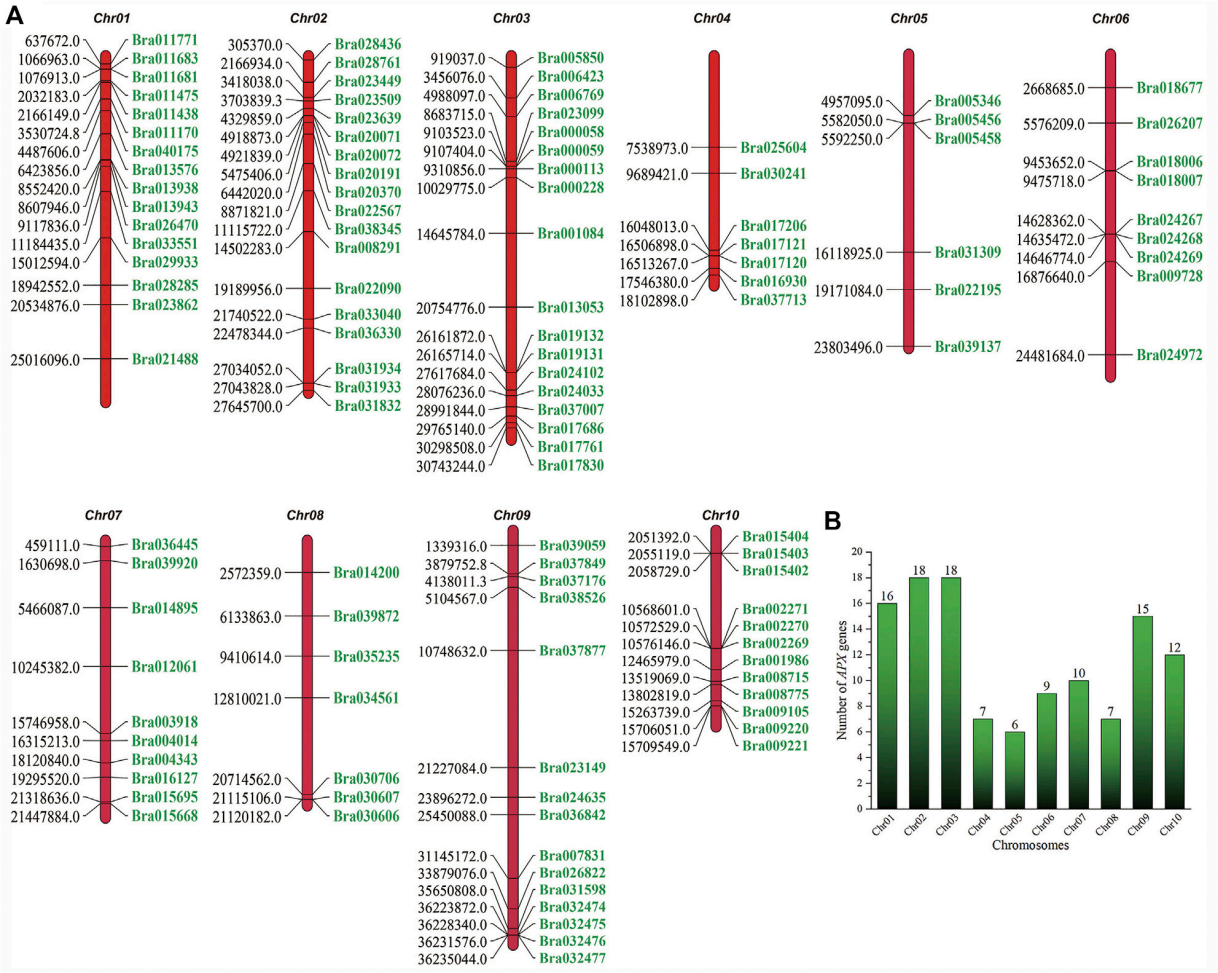
Chromosome mapping of the *APX* genes in *B. rapa*. **(A)** Map of the distribution of *B. rapa APX* genes on 10 chromosomes. The numbers on the left indicate the location of the *APX* genes in *B. rapa* (Mb), and the numbers on the right correspond to the gene number. **(B)** Number of *APX* genes on each chromosome.

The identification of *APX* genes in *B. rapa* and *B. napus* was completed using HMM searches with the aid of the BRAD genome database (version 1.5). These proteins were identified as having the reported peroxidase domains after sequence analysis using the SMART, CDD, Pfam and InterProScan tools. Finally, 118 and 221 candidate *APX* genes were obtained for *B. rapa* and *B. napus*, respectively. BrAPX amino acid residue lengths ranged from 250 aa (Bra030706) to 723 aa (Bra011683), isoelectric point (pI) values ranged from 4.40 (Bra036445) to 10.78 (Bra019132), and molecular weights ranged from 27.39 kDa (Bra017830) to 80.97 kDa (Bra011683) (Supplementary Table S2). We mapped the chromosomal locations of the *APX* genes in *B. rapa* (Figure 1), with most genes located on Chr01 (16 genes), Chr02 (18 genes), Chr03 (18 genes), and Chr09 (15 genes), followed by chromosome Chr10 (12 genes) and Chr04, Chr05, Chr06, Chr07 and Chr08 with seven, six, nine, 10 and seven genes, respectively. These findings indicate that *APX* genes are more widely distributed in the *B. rapa* genome.

**FIGURE 2:**
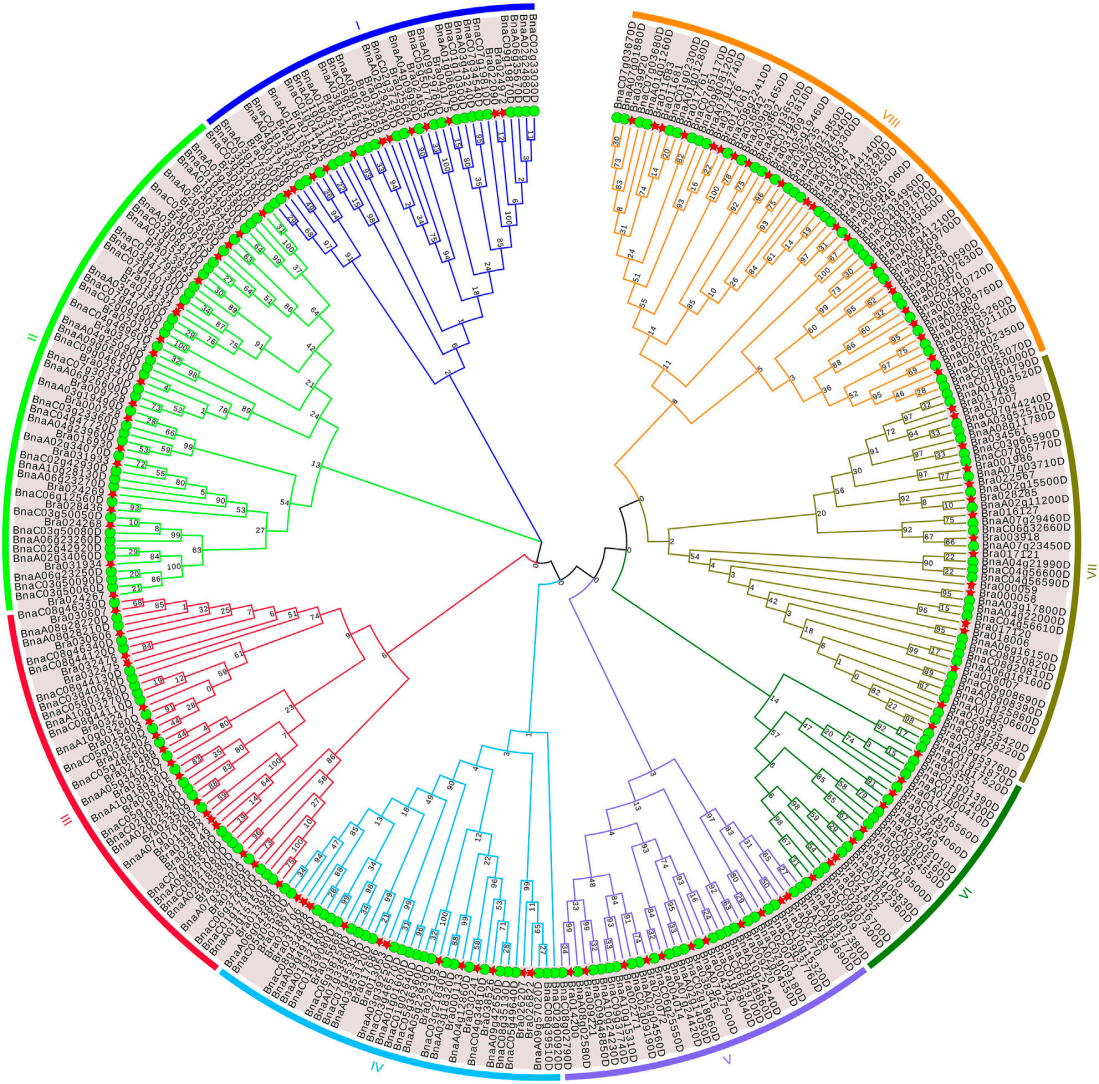
Phylogenetic tree of APX protein of *B.napus* and *B.rapa*. Eight subfamilies and branches are marked with different colors. Green circles represent *B.napus*, and red stars represent *B.rapa*.

**FIGURE 3:**
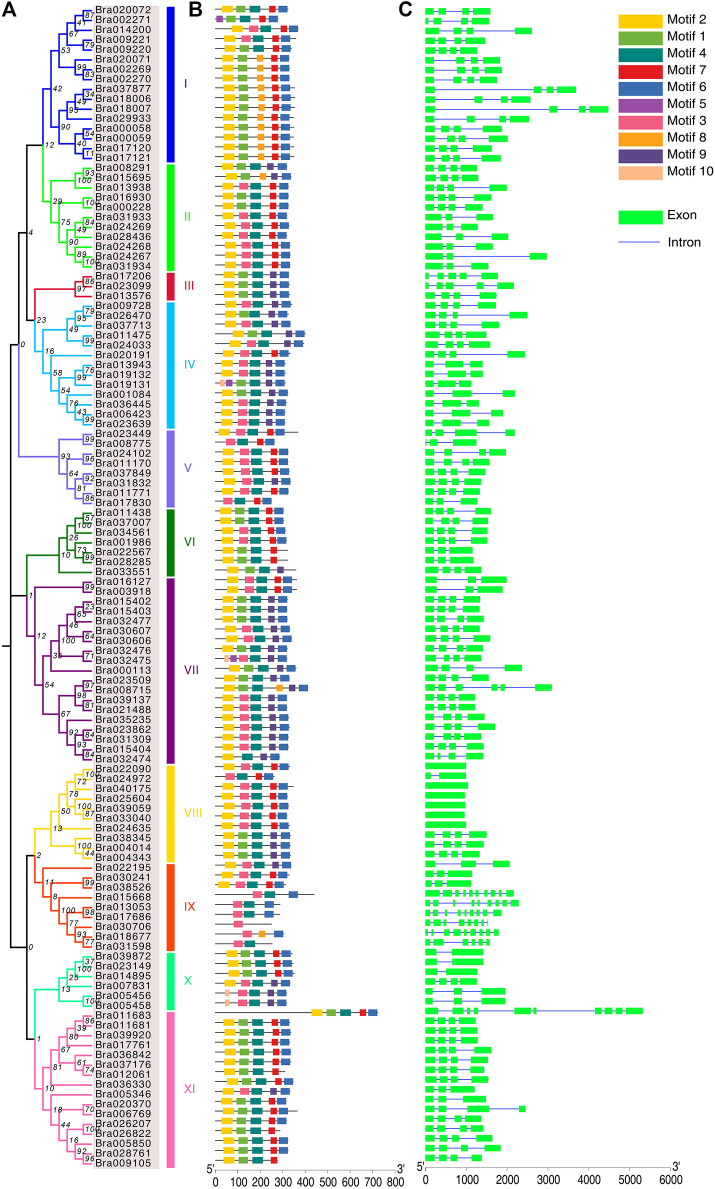
Phylogenetic relationships, gene structure and motif analysis of BrAPXs. **(A)** Phylogenetic tree of BrAPXs; I to XI represent the 11 subgroups. **(B)** Distribution of conserved motifs in BrAPXs. Differently colored boxes represent the 10 conserved domains identified. **(C)** Gene structure of BrAPXs, where green boxes represent exons and blue lines represent introns. The lengths of the boxes and lines are scaled according to the length of the genes.

To further investigate the evolutionary relationship of APX proteins in *B. napus* and *B. rapa*, a rootless maximum likelihood (ML) phylogenetic tree was constructed with APX protein sequences of *B. napus* and *B. rapa* to explore the reasons for their cold resistance (Figure 2). APX was clustered into eight subfamilies (I-VIII) based on sequence similarity and topological structure. Subfamilies II and VIII contain 57 and 58 family members, respectively. Subfamilies I and II are genetically distant from the other subfamilies, and subfamilies V, VI, VII and VIII have closer genetic distances. In addition, we subjected the 118 BrAPX members to unrooted developmental tree construction, dividing these members into 11 subclasses, with cluster III and cluster X containing three and six members (Figure 3A). Our previous studies have shown that the peroxisome pathway is important and complex in the regulation of cold resistance in *B. rapa*, and that APX is able to clear excess intracellular ROS and maintain its homeostasis in a timely manner (Ma et al., 2019a). Therefore, classifying the APX family by phylogeny helps us to clearly explain how the APX family is involved in ROS and cold resistance regulation.

### Gene Structure and Motif Analysis of APX Genes in *B. rapa*

Intron-exon structural models of the *APX* genes were generated through the GSDS server. The APX gene family was found to be diverse in the number of introns and exons, with the number of exons varying from 1 to 10, and the number of introns ranging from 0 to 9 (Figure 3C and Supplementary Table S2). Subfamily IX had the highest number of introns (Bra015668, nine; Bra013053, eight; Bra017686, eight; Bra030706, seven; Bra018677, eight; Bra031598, five), subfamily VIII had the lowest number of introns (Bra039059, 0; Bra033040, 0; Bra025604, 0; Bra024635, 0; Bra040175, 0; Bra022090, 0; Bra024972, one), and subfamily XI contained 10 exons in the gene (Bra011683). The exon-intron structures of these genes distributed in the same cluster are highly conserved.

The MEME server was used to predict the motifs of BrAPX proteins, and a total of 10 widely-distributed conserved motifs were identified (Figure 3B). Motif 2, motif 4, and motif 6 were widely present in 118 APX proteins, with motif 2 containing 50 amino acids, motif 4 containing 48 amino acids, and motif 6 containing 41 amino acids, indicating that these motifs were functionally conserved during evolution (Supplementary Table S3). Additionally, motif 1 and motif 7 were also present in half of the APX proteins, containing 42 and 29 amino acids, respectively. This suggests that these motifs represent conserved motifs and functional domains of the APX protein family in *B. rapa*.

### Gene Duplication and Genomic Collinearity in *B. rapa*.

**FIGURE 4:**
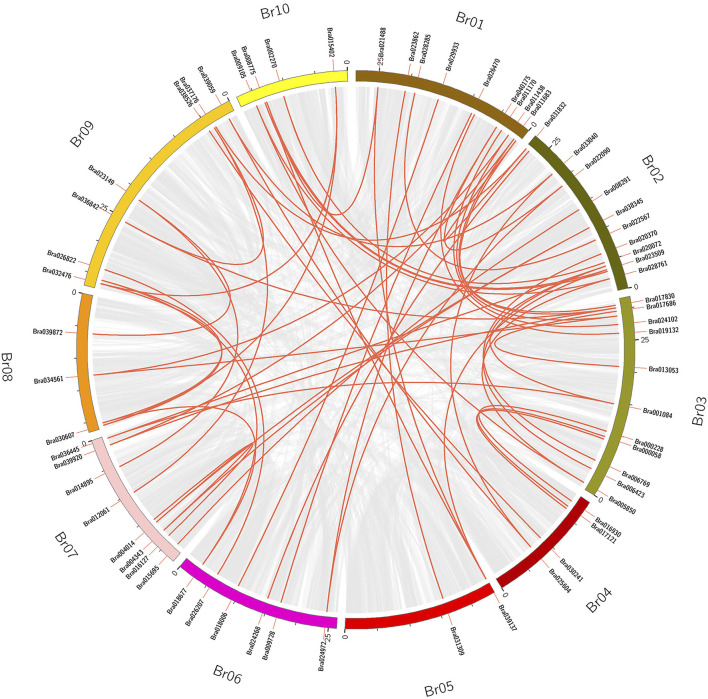
The segmental replication events of BrAPXs in *B. rapa*. Chromosomes are shown in different colors, with all synteny blocks in the *B. rapa* genome shown as grey lines and segmental duplication of *BrAPX* genes shown as red lines, with gene names and chromosome numbers shown outside the diagram.

**FIGURE 5:**
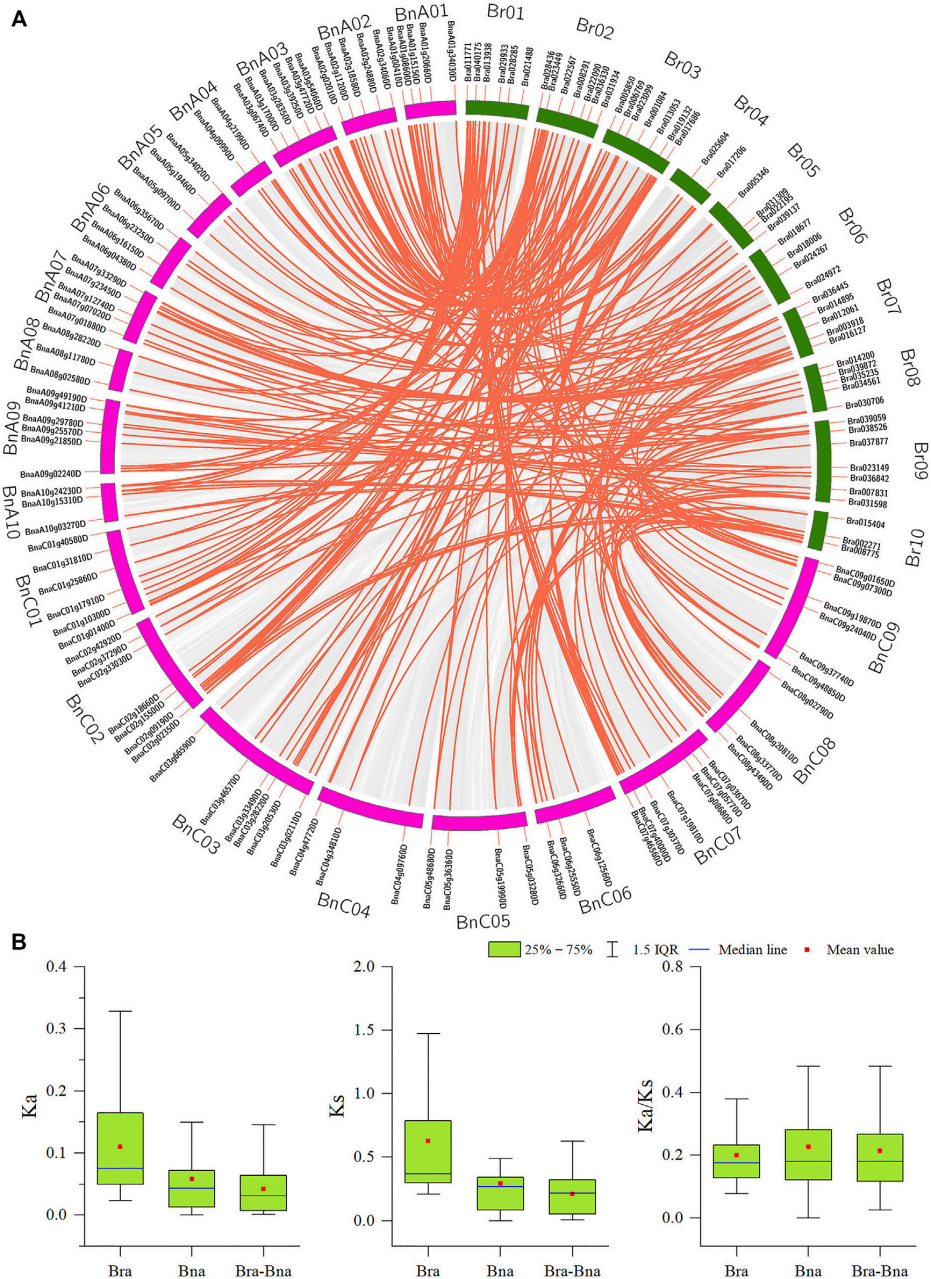
Analysis of orthologous relationships between *APX* gene pairs in *B. rapa* and *B.napus*. **(A)** Circos plot of *APX* gene orthologs in two species. **(B)** Ka, Ks and Ka/Ks values for homozygous *APX* gene pairs between two species. Chromosomes of *B. rapa* and *B. napus* are represented by green and pink bars respectively; The gene number and chromosome label are next to the corresponding chromosome. The red curved lines indicate APX genes with collinearity.

Tandem duplication and segmental duplication events in the *B. rapa* and *B. napus* genomes were studied using MCScanX software (Figures 4, 5A, Supplementary Figure S1 and Supplementary Table S4). No tandem duplication events were identified in the *B. rapa* and *B. napus APX* gene families; subsequently, we identified 62 segmental duplications in *B. rapa* and 193 segmental duplications in *B. napus*, and these segmental duplications were in 90 genes each in the *B. napus* A and B genomes. These results suggest that *APX* genes may have arisen through gene duplication and that segmental duplication events have played an important role in their evolution. To further explore the potential evolutionary processes of the *APX* gene family in *Brassica*, a covariance map of *B. rapa* and *B. napus* was constructed, showing that 268 APX homologous sequences are shared between *B. rapa* and *B. napus*, suggesting that there may be substantial similarities in the molecular functions of *APX* between the two species and that differences in resistance may be due to differences in a few genes. In addition, we calculated Ka/Ks values for *BrAPX* genes, and all pairs of segmentally duplicated *BrAPX* genes had Ka/Ks values < 1, indicating that most of these genes evolved under purifying selection (Figure 5B and Supplementary Table S4).

### Expression of *APX* Genes in *B. rapa* and *B. napus* Under Cold and Freezing Stresses

**FIGURE 6:**
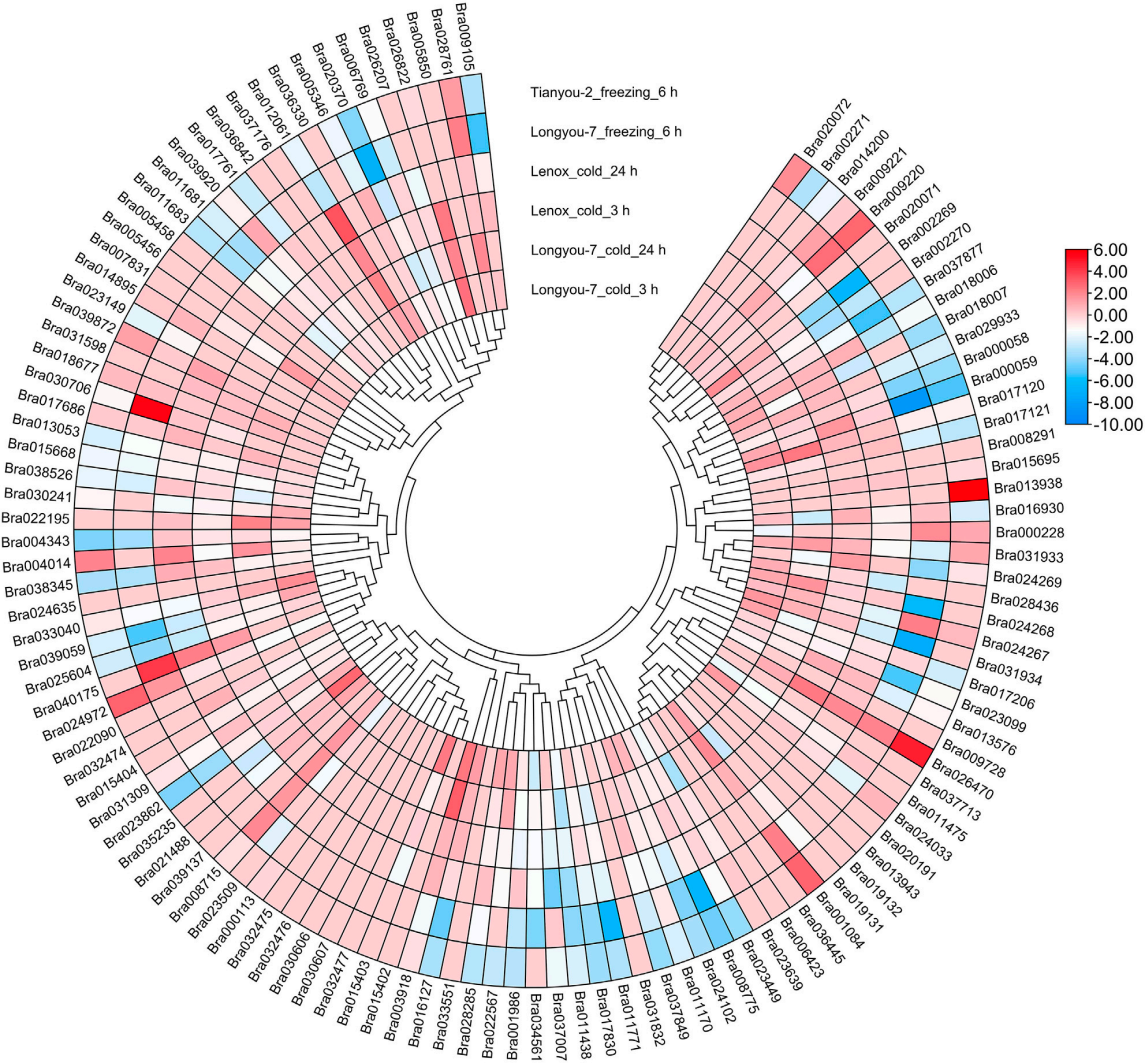
The expression heatmap of *BrAPXs* of different cold-resistant varieties under different stress was plotted according to the log2 mean of FPKM in RNA-seq.

**FIGURE 7:**
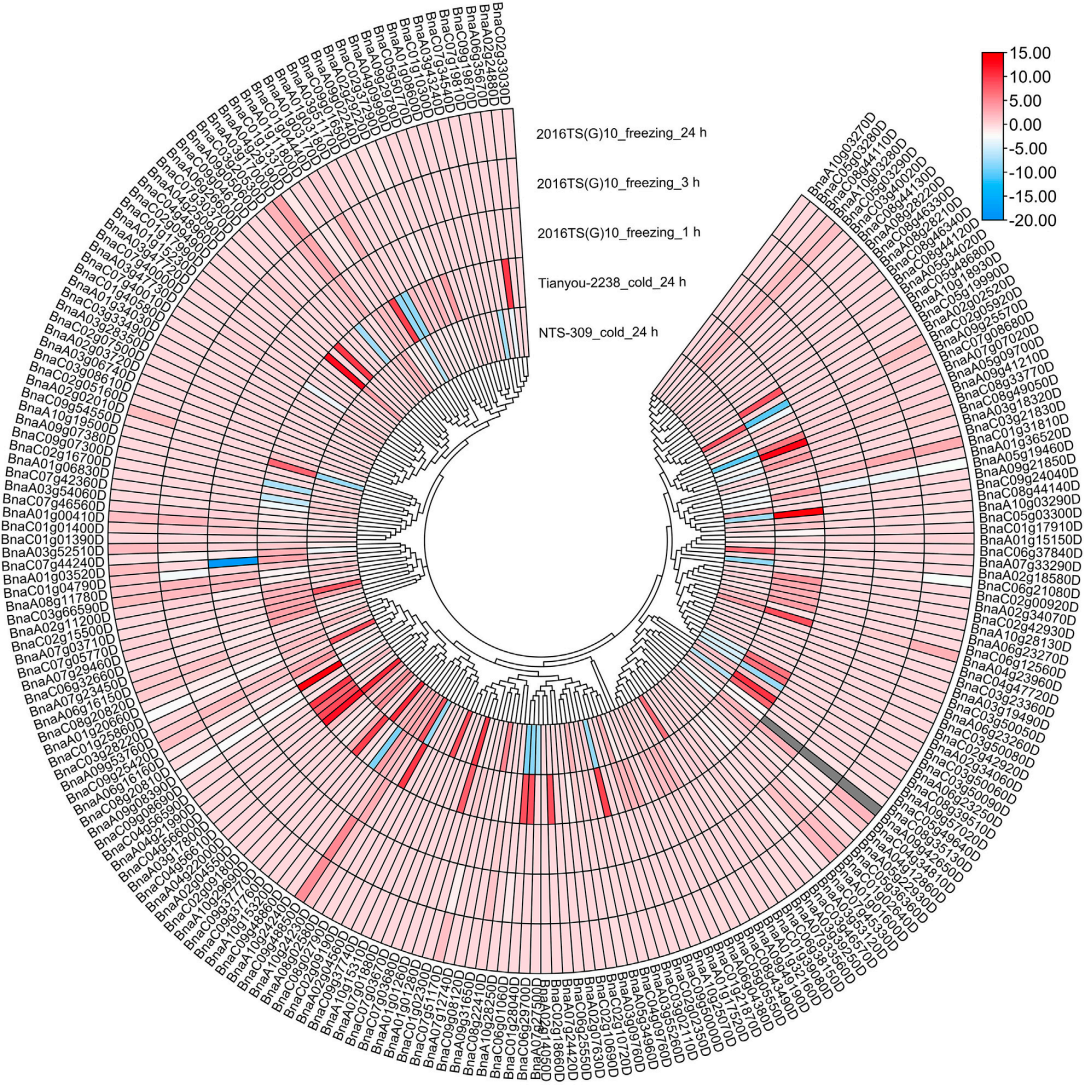
The expression heatmap of *BnAPXs* of different cold-resistant varieties under different stress was plotted according to the log2 mean of FPKM in RNA-seq.

To investigate the expression patterns of *BrAPX* genes under cold stress, RNA-seq was used to analyze expression in growth cones for *B. rapa* varieties differing in cold resistance, and heatmaps were drawn based on their protein evolutionary relationships (Figure 6 and Supplementary Table S5). The results showed that Bra003918, Bra017120, Bra022195, Bra001084 and Bra024268 were highly expressed in cold-tolerant varieties and were more highly expressed at 24 h of cold stress than at 3 h, but were all downregulated in weakly cold-resistant varieties. Expression levels of the four genes (Bra012061, Bra040175, Bra026470, and Bra039872) were higher in the weakly cold-resistant varieties than in the cold-tolerant varieties at 24 h of cold stress. There were 21 and 12 genes significantly upregulated in cold-tolerant varieties at 3 and 24 h under cold stress, respectively, eight and five genes upregulated in weakly cold-resistant varieties at 3 and 24 h under cold stress, respectively. Most of the *BrAPX* genes were downregulated or not significantly expressed. Similarly, we selected RNA-seq expression profiles of cold-tolerant and weakly cold-resistant *B. napus* under cold stress to further analyze the expression patterns of *BnAPX* genes. We used the same method to draw the expression heatmap of *BnAPX* for leaves under cold stress (Figure 7 and Supplementary Table S5). The results showed that the expression of 28 genes was significantly higher in cold-tolerant varieties (“NTS-309”) than in weakly cold-resistant (“Tianyou-2238”) varieties at 24 h of cold stress, and BnaC03g28220D, BnaA02g02520D, and BnaC09g48860D were significantly upregulated in cold-tolerant varieties and significantly downregulated in weakly cold-resistant varieties. The expression of 48 genes was significantly higher in weakly cold-resistant varieties than in cold-tolerant varieties at 24 h of cold stress, and 38 of these genes were significantly upregulated in weakly cold-resistant varieties and significantly downregulated in cold-tolerant varieties. In addition, we found that most *APX* genes were downregulated in *B. napus*.

We analyzed the RNA-seq data of *B. rapa* under freezing stress. There were 21 and 18 upregulated expressed genes and 51 and 55 downregulated expressed genes in the cold-tolerant and weakly cold-resistant varieties, respectively, under the -4°C for 6 h treatment. The expression trend of *BrAPX* gene family in two varieties with different cold resistance is the same. Under freezing stress, most of the *BrAPX* genes were downregulated in roots. The expression trend in *B. napus* was that the expression of upregulated genes in its leaves increased gradually with increasing freezing stress time, while the expression of downregulated genes did not differ significantly at different treatment times. There were 45 upregulated genes, 33 downregulated genes, and 143 non-expressed genes (Figures 6, 7 and Supplementary Table S5). The *BnAPX* gene family was not actively expressed under freezing stress.

**FIGURE 8:**
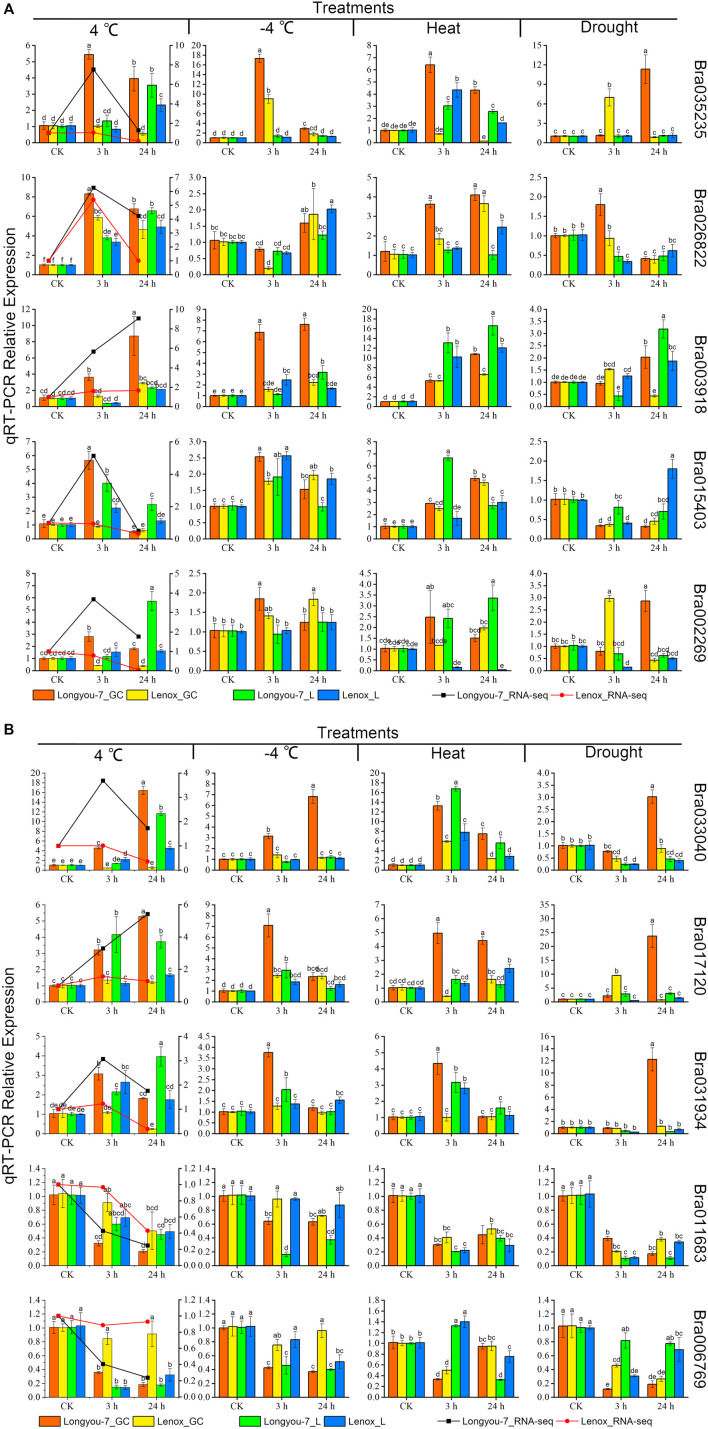
Ten *APX* genes were analyzed by qRT-PCR expression in growth cones (GC) and leaves (L) of cold-tolerant (“Longyou-7”) and weakly cold-resistant varieties (“Lenox”) under cold, freezing, drought, and heat stress treatment **(A and B)**. Different columns represent four different abiotic stresses, and each row shows the expression of the same gene under different treatments. The line graph indicates the FPKM values of the growth cones under 4°C treatment. Gene expression was normalized to the expression level of normal growth at room temperature and assigned a value of 1. Data represent the mean ± standard error for three biological experiments, with standard errors shown as bar charts above the columns, while lowercase letters indicate significance at *p* < 0.05.

We selected 10 differentially expressed *BrAPX* genes from the RNA-seq data for qRT-PCR analysis. The qRT-PCR expression pattern of *BrAPX* genes in *B. rapa* growth cones under cold stress was consistent with the RNA-Seq dataset with high linearity (y = 0.851x - 0.17, *R* ^*2*^ = 0.876) (Figures 8A,B, Supplementary Figure S2). Bra035235, Bra026822, Bra015403, Bra002269 and Bra031934 were significantly upregulated in growth cones at 3 h of cold stress, while their expression was significantly lower at 24 h than at 3 h. Among them, Bra035235 expression in “Longyou-7” growth cones was 4.1, 6.6, and 5.4 times higher than that in “Longyou-7” leaves, “Lenox” leaves, and roots, respectively, under 3 h treatment. At 24 h treatment it was 7.1 times higher than in “Lenox” growth cones. Bra015403 was significantly more highly expressed in “Longyou-7” growth cones than in other treatments, being 6.4-fold higher than in “Lenox” growth cones at the 3 h treatment. Their expression was higher in cold-tolerant than in weakly cold-resistant varieties, indicating that these genes are the first to be activated at the onset of cold stress and that they are stimulus genes. Bra003918, Bra033040, Bra017120, Bra011683 and Bra006769 were continuously expressed in growth cones with the increasing duration of cold stress. The expression of Bra003918 in “Longyou-7” growth cones was significantly higher than the others at 3 and 24 h treatments, and 2.9 and 3 times higher than in “Lenox” growth cones, respectively. Bra033040 expression in “Longyou-7” growth cones was 10.8 and 34.5 times higher than in “Lenox” growth cones at 3 and 24 h treatments, respectively. Bra017120 expression in “Longyou-7” growth cones was 2.4 and 4.4 times higher than in “Lenox” growth cones under 3 and 24 h treatments, respectively. In leaves, Bra035235, Bra026822, Bra003918, Bra002269, Bra033040, Bra031934, Bra011683 and Bra006769 were continuously upregulated under cold stress, and the expression at 24 h was significantly higher than that at 3 h. Consistent with the growth cones, the expression in cold-tolerant varieties was higher than that in weakly cold-resistant varieties. Bra002269 and Bra031934 were the most highly expressed in “Longyou-7” leaves, with 3.5- and 2.3-fold more expression than in “Lenox” leaves under 24 h treatment, respectively. In addition, we found that Bra003918, Bra015403, Bra033040, Bra011683, and Bra006769 showed similar expression patterns in growth cones and leaves.

### Expression of *APX* Genes in *B*. *rapa* Against Heat, Drought and Freezing Stresses

**FIGURE 9:**
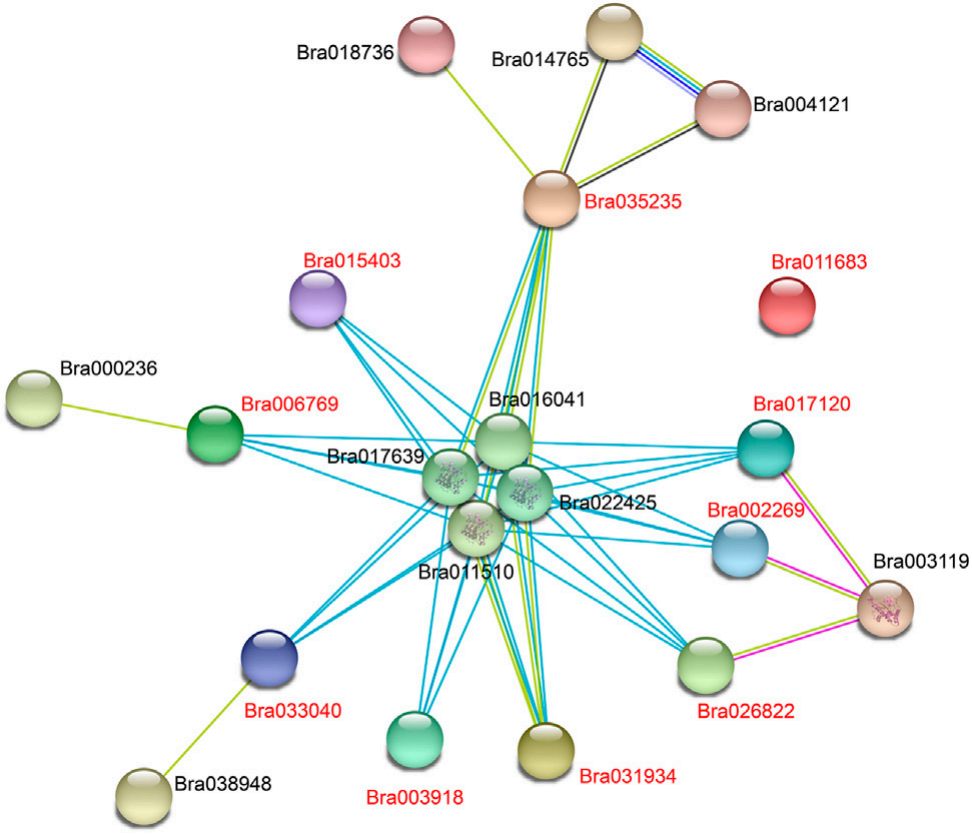
Predicting interactions between BrAPXs using STRING software (Szklarczyk et al., 2021). The 10 BrAPX proteins screened are labelled in red. Network nodes represent proteins, and edges represent protein-protein associations. Light blue lines represent known interactions from curated databases, pink lines represent known interactions determined experimentally, blue lines represent gene co-occurrence, yellow lines represent text mining, and black lines represent co-expression.

To further investigate the expression patterns of these 10 *APX* genes differentially expressed under cold stress under other abiotic stresses, we performed qRT-PCR analysis on these two varieties under heat (40°C), PEG (PEG6000, 18%), and freezing (−4°C) stress conditions for 3 and 24 h (Figures 8A,B). Five *APX* genes (Bra035235, Bra003918, Bra033040, Bra017120, and Bra031934) were significantly expressed in cold-tolerant varieties under freezing stress and were more highly expressed than in weakly cold-resistant varieties. Bra035235 and Bra017120 were expressed in “Longyou-7” growth cones at levels 2 and 2.9 times higher than “Lenox” growth cones at −4°C under 3 h treatment, respectively. The expression of Bra003918 and Bra033040 in “Longyou-7” growth cones was 4.3, 3.4, 2.2, and 6 times higher than in ‘Lenox’ growth cones at −4°C for 3 and 24 h treatments, respectively. The expression of these genes was higher in growth cones than in leaves, suggesting that cold-tolerant varieties of growth cones have an important role in cold resistance. When leaves wilted after the cold overwintering period in northern China, these results were also shown during cold stress. After 24 h of simulated drought stress, Bra035235, Bra002269, Bra033040, Bra017120 and Bra031934 were significantly upregulated in “Longyou-7” growth cones, at levels 13.4, 6.6, 3.4, 32.9, and 10 times that of “Lenox” growth cones, respectively. The expression of Bra003918 and Bra015403 was higher in “Longyou-7” leaves than in growth cones under drought stress. Under heat stress, Bra035235, Bra003918, Bra015403, Bra033040 and Bra031934 were significantly upregulated in growth cones and leaves of both species, with higher expression in “Longyou-7” than in “Lenox”. The expression of Bra017120 in “Longyou-7” growth cones was 12.2 and 2.7 times higher than that in “Lenox” growth cones at 3 and 24 h, respectively. The results showed that *B. rapa* leaves were more susceptible to heat stress, and cold-tolerant varieties may additionally have had stronger heat tolerance. We performed interaction analysis on the 10 genes screened, a total of 90 pairs of interacting proteins were predicted. All nine proteins interacted with Bra011510, Bra016041, Bra017639, and Bra022425, except for Bra011683. There were 14 pairs of proteins that interacted with Bra035235 and 10 pairs of proteins that interacted with Bra017120. In addition, these genes are involved in plant peroxidase, plant methyltransferase dimerization, and phenylpropanoid biosynthesis pathways (STRING 11.5 software) (Figure 9 and Supplementary Table S6). In summary, the Bra035235, Bra003918, Bra033040, Bra017120, and Bra031934 genes are closely related to the abiotic stress response in *B. rapa* and deserve further study.

## Discussion

APX (EC, 1.11.1.11) belongs to the peroxidase (PF00141) family, and is considered to be a key enzyme in H_2_O_2_ elimination. It also plays a role in plant growth and development as well as adversity response (Gilroy et al., 2016; Tao et al., 2018). The detoxification of H_2_O_2_ by APX is a series of reactions catalyzed by monodehydroascorbate reductase (MDHAR), dehydroascorbate reductase (DHAR), and glutathione reductase (GR). The ascorbate-glutathione or Halliwell-ASADA cycle, which combines these processes, is one of the most important antioxidant systems in plants. Ascorbate and glutathione are employed as reducing substrates for scavenging of H_2_O_2_ in this cycle, and they are ultimately cycled at the expense of ATP and NAD(P)H (Teixeira et al., 2006). Although APX is known to be involved in a variety of abiotic stress-related responses, the APX gene family in *B. rapa* has yet to be fully characterized.

In this work, 118 *APX* genes were identified in *B. rapa,* and comparisons with the *B. napus* APX gene family revealed that the A genome contained much less APX members than the AC genomes, indicating that APX is closely linked evolutionarily between these two species. In the *B. rapa APX* gene family, there have been no tandem duplication events, but there have been 62 segmental duplication events, with the majority of the *BrAPX* gene segmental duplications localized on chromosomes A1, A2, and A3. In *B. napus*, we also found greater numbers of segmental duplications on chromosomes A1, A2, and A3. This suggests that segmental duplication events have played an important role in the evolution of the *BrAPX* genes. The research showed that all of *Brassica* underwent an extra whole genome triplication (WGT) event that occurred approximately 9–15 million years ago, or even approximately 28 million years ago (Lukens et al., 2004; Arias et al., 2014). After the WGT event, the ASA content and the number of expressed genes did not increase significantly with the increase of ASA-related genes in rape. To protect rape from oxidative stress it retains a number of ASA-related genes, and these unexpressed ASA genes may play an alternative role in response to severe abiotic stresses (Duan et al., 2014). Tandem duplication and segmental duplication contribute to the expansion of new gene family members and novel functions in the evolution of plant genomes (Wei et al., 2019; Li et al., 2020). As a result of duplication events, some members have been able to evolve to acquire new functions to enhance their resistance or have become pseudogenes and lost their original functions (Dias et al., 2003). Thus, the WGT in *B. rapa* play a positive role in the evolution of the APX gene family.

These APX members of *B. rapa* were divided into 11 subfamilies that showed diversity in the number of introns and exons, relative concordance in number between subclusters, and most members contained motif 2, motif 4 and motif 6 conserved structural domains, reflecting the functional richness of the APX gene family under multiple stresses. In addition, low homology and functional domains were found in the different APX subfamilies. This indicates that the *BrAPX* families are highly divergent in origin and functional evolution. The 26 *GhAPX* genes identified in cotton were classified into five subfamilies, and the homology of the different APX subfamilies was low (Tao et al., 2018). In another study, a *sorghum*-*Arabidopsis* APX tree of 17 sequences was constructed using known *Arabidopsis* APX sequences, with different clusters of APX protein having different subcellular localizations, and inferring that *sorghum* APXs has similar functions to its *Arabidopsis* homologs in the same cluster (Tognolli et al., 2002; Akbudak et al., 2018). Based on these clustering topologies, we speculate that APX in the same cluster of *B. rapa* and its *B. napus* homologs in have similar functions.

Our previous studies have shown that the expression of peroxisome pathway genes in different cold-resistant varieties of *B. rapa* differs under cold stress, and these differentially expressed genes are closely related to cold resistance (Ma et al., 2019a; Qi et al., 2020). This study further analyzed the expression of the *APX* gene family under cold stress. At 3 and 24 h of cold stress, Bra003918, Bra017120, Bra022195, Bra001084, and Bra024268 were significantly upregulated in the cold-tolerant variety “Longyou-7”, with Bra003918 3.71 and 4.32 times more highly expressed than in the weakly cold-resistant variety “Lenox” at 3 and 24 h, respectively (log2–fold), whereas it was not significantly expressed in Lenox (Figure 6 and Supplementary Table S5). We also found that 21 of the 45 upregulated genes were significantly expressed in cold-tolerant varieties at 3 h of cold stress, and most of them were expressed more than in weakly cold-resistant varieties. This suggests that *APX* of *B. rapa* is activated early in cold stress, allowing cold-tolerant varieties to establish defenses against H_2_O_2_ as early as possible (Panchuk et al., 2005; Guan et al., 2015). In addition, we found that upregulated genes were distributed across eight subclusters, with subclusters VII and XI having a higher number of upregulated genes. Expression of members of subfamilies V and VI was downregulated under cold stress, and the same expression trend was observed under freezing stress. Although the downregulated genes in both stresses were slightly different among individuals, the number of downregulated genes was similar, and the number of differentially downregulated genes was much greater than that of upregulated genes. Which may have been related to the functional diversity of the rape *APX* family and the ability of these plants to cope with different environmental challenges (Verma et al., 2019). Teixeira et al. (2006) revealed that antioxidant defense systems between different cellular are complementary and coordinated during developmental and abiotic stresses. Therefore, we consider that the functions of Bra003918, Bra017120, Bra022195, Bra001084, and Bra024268 genes under cold and freezing stress deserve to be further investigated.

Akbudak et al. (2018) identified nine APX members and seven GPX members in the *sorghum* genome, and RNA-seq data indicated that *APX* was relatively more responsive to drought stress in sensitive genotypes compared to *GPX*, and that expression of *APXS*/*GPXs* was upregulated in leaves and downregulated in roots under drought stress, with most differentially expressed genes were located in chloroplasts, mitochondria, and peroxisomes. It is also suggested that the main reason for the decrease in *APX*/*GPX* expression in *sorghum* roots may be changes in the source-sink relationship, and that these differentially expressed genes may play an important role in alleviating drought stress in *sorghum*. Other studies have shown that *APX* transgenic plants of *Arabidopsis*, potato, sweet potato, and cotton generally have elevated APX content and an increased ability to eliminate reactive oxygen species during abiotic stresses (Guan et al., 2015; Yan et al., 2016; Shafi et al., 2017; Zhou et al., 2021). In the current study, we found that Bra035235, Bra017120 and Bra031934 genes were significantly upregulated under four abiotic stress conditions, and this upregulation trend was more prominent in cold-tolerant varieties. Furthermore, expression of individual genes showed opposite trends under different abiotic stresses. While other genes were only expressed under a single abiotic stress. This indicates the complexity of *B. rapa* APXs’ response to abiotic stresses. The harsh ecological conditions in northern China have caused the whole growth period of *B. rapa* to be affected by various abiotic stresses such as cold, drought, salinity, and high temperature, indicating that widely adapted varieties will show better resistance than other types. Therefore, *APX* genes that simultaneously respond to multiple abiotic stresses deserve our in-depth attention.

## Conclusion

In summary, we identified 118 *BrAPX* genes from *B. rapa* and positioned them unevenly on 10 chromosomes. These BrAPX proteins were classified into 11 subclasses and were closely associated evolutionarily with *B. napus*, with segmental replication events playing a major role. Three conserved motifs are widely present in these proteins. Five key genes (Bra035235, Bra003918, Bra033040, Bra017120 and Bra031934) responsive to abiotic stress were found. These genes are closely related to the abiotic stress response of *B. rapa*, which is worthy of further study. In addition, we have identified a number of candidate genes (Bra003918, Bra017120, Bra022195, Bra003918, Bra033040, BnaC09g48850D, BnaC03g28220D, BnaA02g02520D, and BnaC09g48860D) that may play important roles in the response of *B. rapa* and *B. napus* to cold and freezing stresses. This work provides new ideas for studying the involvement of the *APX* gene family in the molecular mechanisms of cold resistance in *B. rapa*.

## Data Availability

The datasets presented in this study can be found in online repositories. The names of the repository/repositories and accession number(s) can be found in the article/Supplementary Material.

## Footnotes

1
http://pfam.xfam.org/

2
http://brassicadb.org/brad/index.php/

3
http://hmmer.org/download.html/

4
http://smart.embl-heidelberg.de/

5
http://www.ncbi.nlm.nih.gov/Structure/cdd/wrpsb.cgi/

6
http://web.expasy.org/protparam/

7
http://alternate.meme-suite.org/tools/meme/

8
http://gsds.cbi.pku.edu.cn/

9
http://chibba.pgml.uga.edu/mcscan2/

10
https://sourceforge.net/projects/kakscalculator2/

11
https://github.com/CJ-Chen/TBtools

12
http://string-db.org/

## Supplementary Material

Supplementary Figure S1 The segmental replication events of BnAPXs in B. napus.

Supplementary Figure S2 Correlation between RNA-seq FPKM values and qRT-PCR expression.

## Abbreviations

B. rapa, Brassica rapa; B. napus, Brassica napus; APX, Ascorbate peroxidase; BrAPX, Ascorbate peroxidase gene in Brassica rapa; BnAPX, Ascorbate peroxidase gene in Brassica napus; qRT-PCR, Quantitative real-time-PCR; HMM, Hidden Markov model; Ks, Synonymous substitution rate; Ka, Non-synonymous substitution rate.
