# Supplementary material for: Genome-Wide Identification and Analysis of the Ascorbate Peroxidase (APX) Gene Family of Winter Rapeseed (Brassica rapa L.) Under Abiotic Stress
Source: Front Genet. 2022 Jan 21;12:753624. doi: 10.3389/fgene.2021.753624 (PMC8814366; doi:10.3389/fgene.2021.753624)
Supplement: Supplementary file 3 [file Table1.DOCX]

**Table S1 Primer sequence of qPCR**

| Gene ID | Sequence(5'to3') (forward/reverse) | Amplicon length (bp) | qPCR efficiency (%) |
| --- | --- | --- | --- |
| Bra035235 | AGACTGTGCTGGAGAGGAAGTGTC/ TCCGTCTCTGCGTCCTGTTGG | 128 | 103 |
| Bra026822 | TGGCAAACTTTCAGTCAGCGAGAG/ GTCTCTAGCGGCAACGGCAATG | 119 | 98.7 |
| Bra003918 | TCAGCGAGTGGACCAGGAGAAC/ TGCGGCTAGAGCGAGGATGTC | 147 | 107.2 |
| Bra015403 | CTACAAGCACAGGTGTCCAGACG/ CGGCAAGACTTGGCTCGTGAG | 83 | 102.1 |
| Bra002269 | TGCCCTGGAGTCGTCTCTTGTG/  GCCGTGCGACCATCTCTTCTTC | 116 | 108.1 |
| Bra033040 | TCCTCATTCTCCTCGCCTCCAC/ GGTCGGGTTGGTGATTTGTTTGC | 125 | 103.9 |
| Bra017120 | TCCGAGAAAGATGCTGCTCCAAAC/ ACATCCGCACAAGACACTGTTCC | 113 | 109.8 |
| Bra031934 | TCTTCAACTTCAACGGCACAGGAC/ ACTCGGGCTGATGCGTCTCC | 106 | 101.7 |
| Bra011683 | CATCGCCGCAAAGAAGCAGATTG/ CAACCGCCTGAAGACGAGTAACC | 116 | 105.6 |
| Bra006769 | GCTCGTGACTCTGTTCTCCTCTTG/ TGAAACTCGCTGTGGTGGAATCTC | 82 | 104.7 |
| β-actin | TGTGCCAATCTACGAGGGTTT/  TTTCCCGCTCGGCTGTTGT | 137 | 98.4 |
