# Supplementary figures and images for: Genome-Wide Identification and Analysis of the Ascorbate Peroxidase (APX) Gene Family of Winter Rapeseed (Brassica rapa L.) Under Abiotic Stress
Source: Front Genet. 2022 Jan 21;12:753624. doi: 10.3389/fgene.2021.753624 (PMC8814366; doi:10.3389/fgene.2021.753624)

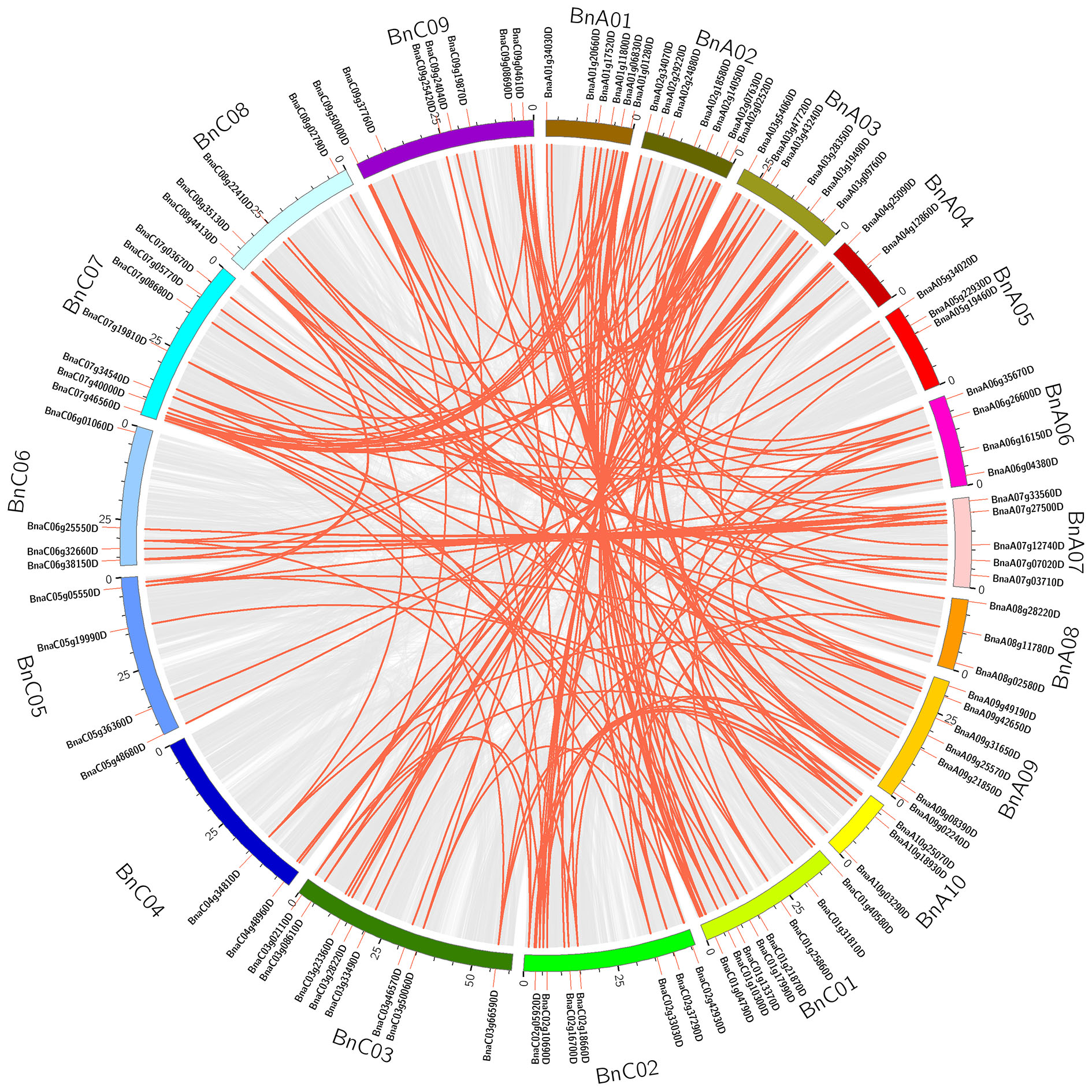

Supplement: Supplementary file 4 [file Image1.JPEG]

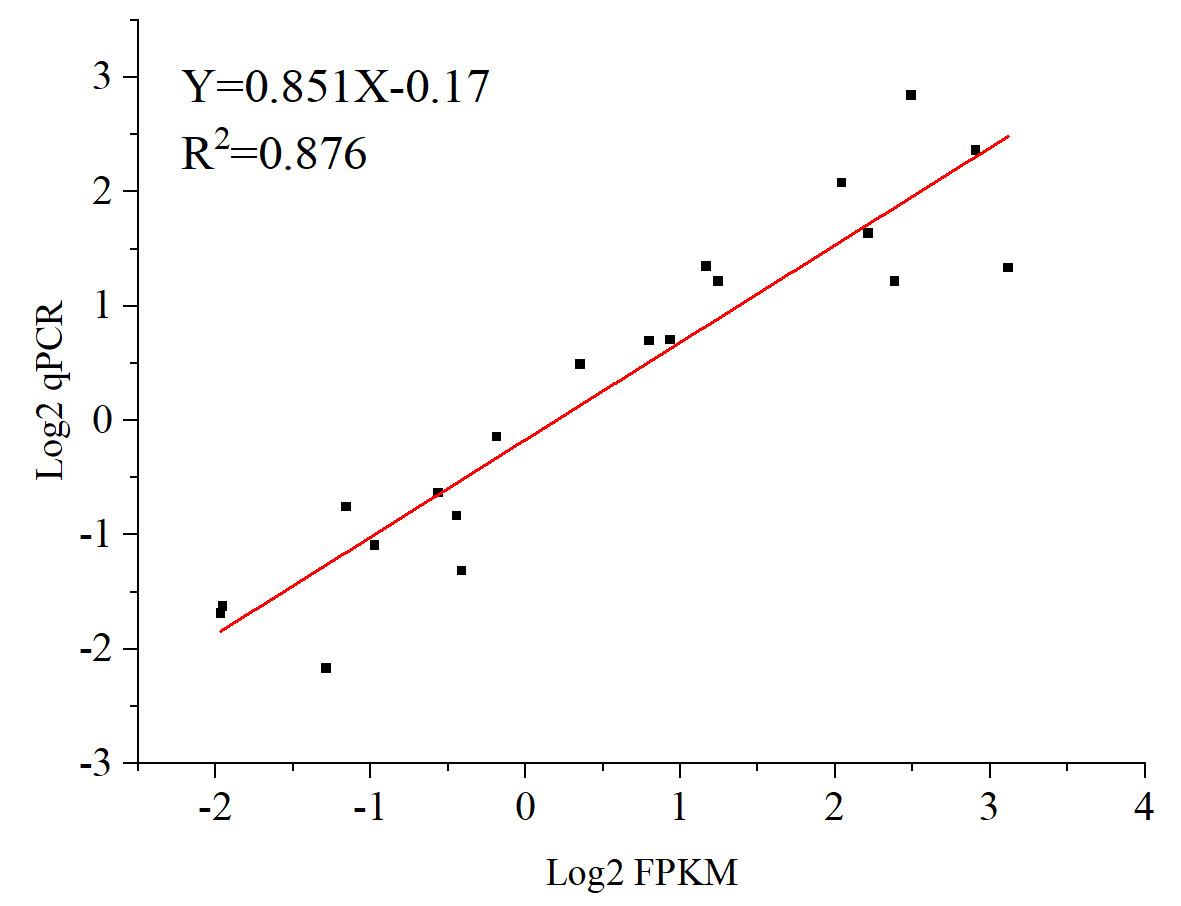

Supplement: Supplementary file 5 [file Image2.JPEG]
